# The Enhanced Performance of Neuromorphic Computing Hardware in an ITO/ZnO/HfO_x_/W Bilayer-Structured Memory Device

**DOI:** 10.3390/nano13212856

**Published:** 2023-10-28

**Authors:** Minseo Noh, Dongyeol Ju, Seongjae Cho, Sungjun Kim

**Affiliations:** 1 Division of Electronics and Electrical Engineering, Dongguk University, Seoul 04620, Republic of Korea; 021015min@gmail.com (M.N.);; 2 Department of Electronic and Electrical Engineering, Ewha Womans University, Seoul 03760, Republic of Korea

**Keywords:** neuromorphic system, resistive switching, ZnO, spike-timing-dependent plasticity

## Abstract

This study discusses the potential application of ITO/ZnO/HfO_x_/W bilayer-structured memory devices in neuromorphic systems. These devices exhibit uniform resistive switching characteristics and demonstrate favorable endurance (>10^2^) and stable retention (>10^4^ s). Notably, the formation and rupture of filaments at the interface of ZnO and HfO_x_ contribute to a higher ON/OFF ratio and improve cycle uniformity compared to RRAM devices without the HfO_x_ layer. Additionally, the linearity of potentiation and depression responses validates their applicability in neural network pattern recognition, and spike-timing-dependent plasticity (STDP) behavior is observed. These findings collectively suggest that the ITO/ZnO/HfO_x_/W structure holds the potential to be a viable memory component for integration into neuromorphic systems.

## 1. Introduction

The advancement of modern computing and information storage technology hinges on innovations in memory technology. Due to the scaling limits of current technology, Moore’s law is coming to an end [1]. Furthermore, to address the potential von Neumann bottleneck inherent in the traditional CPU-memory separation architecture, researchers are exploring the integration of various memory devices into neuromorphic computing systems, including phase-change RAM (PcRAM) [2,3], ferroelectric random-access memory (FRAM) [4,5], magnetic RAM (MRAM) [6,7], and resistive RAM (RRAM) [8,9]. Attention has shifted toward alternatives that break from conventional evolutionary paths. Among these, RRAM has garnered prominence as a promising contender for the next generation of non-volatile memory [10,11]. Its appeal lies in its straightforward metal–insulator–metal (MIM) structure, rapid speed, compatibility with CMOS technology, and low power consumption [12,13,14,15,16,17,18,19,20,21,22,23,24,25]. This innovative approach aims to bridge the gap between CPU and memory, promising efficient data processing and potentially revolutionizing the computing landscape. Operationally, RRAM has a simple structure and operation. By applying voltage to the electrodes, the device switches between a high-resistance state (HRS) and a low-resistance state (LRS), forming the foundation for data storage [26]. This resistive switching phenomenon occurs within insulating materials such as HfO_x_ [27,28], ZnO [29], Al_2_O_3_ [30], NiO [31], and others. They can be deposited using various techniques, including chemical vapor deposition (CVD) and physical vapor deposition (PVD). Among these materials, ZnO-based RRAM stands out for synaptic device applications due to its notable features, including a moderate bandgap and cost-effectiveness [32]. Nevertheless, challenges persist within single-layer ZnO RRAM, encompassing critical switching variations, power consumption concerns, non-uniform SET and RESET voltages, and the presence of high-leakage currents [33].

Jain et al. reported that HfO_x_ showcases a lower Gibbs free energy and a higher dielectric constant compared to ZnO, resulting in the formation of fewer oxygen vacancies. The interface between ZnO and HfO_x_ promotes the creation and rupture of filaments, enhancing endurance stability by preventing the escape of oxygen vacancies from the electrode [34,35,36]. This insight suggests that incorporating HfO_x_ in RRAM devices contributes to improved operational stability [37,38,39]. Building upon this foundation, our research introduces an innovative methodology, integrating HfO_x_ to establish a dependable conductive filament and facilitate the exploration of synaptic behaviors. Our investigation addresses the challenge of achieving high switching efficiency and stability, presenting an advanced framework for advancing RRAM technology. Moreover, our study goes beyond material properties and filament stability, instead aiming to replicate the progressive conductivity changes resembling the human neural network. We employ excitatory post-synaptic current and spike-timing-dependent plasticity and emulate the human brain using the Modified National Institute of Standards and Technology (MNIST) database [40,41,42,43,44]. In this work, we delve into the progress and challenges of RRAM technology, focusing on enhancing neuromorphic RRAM devices through integrating HfO_x_. Investigating improved properties, including synaptic behavior, we aim to contribute to the advancement of next-generation non-volatile memory and computing systems.

## 2. Experimental Section

To ensure a clean surface, a commercially available SiO_2_/Si substrate was cleaned using acetone and isopropyl acetone. The bottom W electrode was deposited through a radio frequency (RF) sputter process with 20 sccm of Ar gas. The pressure and RF power were 399.966 mPa and 100 W. For the ZnO/HfO_x_ bilayer device, a 5 nm thick HfO_x_ film was deposited on the bottom W electrode through an RF reactive sputter using a gas mixture of Ar (12 sccm) and O_2_ (3 sccm) with RF power of 80 W. For both devices, a 30 nm thick ZnO film was deposited through an RF reactive sputter (at 80 W) in a gas mixture of Ar (12 sccm) and O_2_ (8 sccm). Using photolithography, square patterns of 100 µm × 100 µm were made on the ZnO films. Finally, a 100 nm thick top ITO electrode was acquired through an RF sputtering process (at 60 W) using Ar gas (8 sccm). A commercial ITO target (99.99% purity) was used under 399.966 mPa gas pressure. The cross-section analysis and its chemical properties were acquired using a field emission transmission electron microscope (JEOL JEMF200, Akishima, Japan) and X-ray photoelectron spectroscopy (XPS). Furthermore, the electrical properties of ITO/ZnO/HfO_x_/W and ITO/ZnO/W devices were investigated using a semiconductor parameter analyzer (Keithley 4200-SCS and 4225-PMU ultrafast module, Cleveland, OH, USA) by biasing ITO top electrode, leaving W electrode grounded.

## 3. Results and Discussions

**Figure 1:**
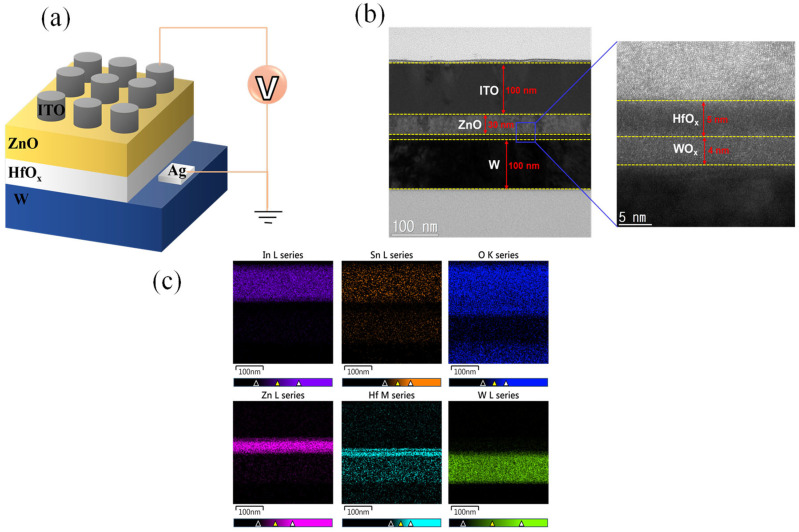
(**a**) Schematic illustration of ITO/ZnO/HfO_x_/W device. (**b**) Cross-sectional TEM image of the ITO/ZnO/HfO_x_/W device. (**c**) EDS line spectra of In, Sn, O, Zn, Hf, W.

Figure 1a illustrates the schematic representation of the ITO/ZnO/HfO_x_/W device. The square-patterned top electrode with a diameter of 100 μm was achieved through photolithography and a lift-off process using acetone. In Figure 1b, we present cross-sectional transmission electron microscopy (TEM) images of the ITO/ZnO/HfO_x_/W device. These images distinctly depict a 30 nm ZnO layer, a 100 nm top ITO electrode, a bottom W electrode, and a 5 nm HfO_x_ layer. Intriguingly, we also observe the presence of a 4 nm WO_x_ layer, which formed as a result of oxygen migration from HfO_x_. Figure 1c further complements our analysis by showcasing energy-dispersive X-ray spectroscopy (EDS) line spectra for each layer, affirming the presence of In, Sn, O, Zn, Hf, and W.

**Figure 2:**
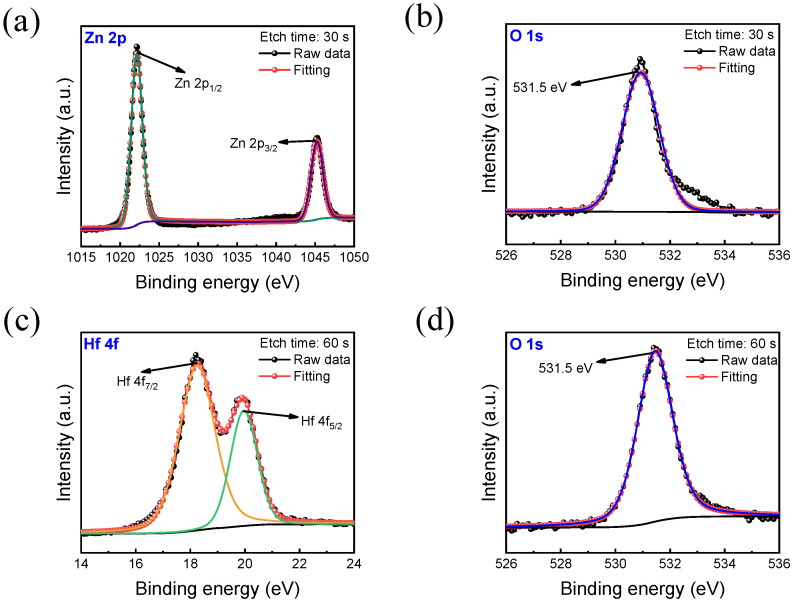
The XPS spectra of ZnO and HfO_x_ thin films. (**a**) Zn 2P. (**b**) O 1s. (**c**) Hf 4f. (**d**) O 1s.

Further chemical compositions of the insulating HfO_x_ and ZnO layers were conducted through X-ray photoelectron spectroscopy (XPS) analysis. Figure 2 provides an insight into the chemical composition of the RF-sputter-deposited ZnO/HfO_x_ bilayer film.

Firstly, Figure 2a,b showcase the XPS spectra for Zn 2p and O 1s in the ZnO film. As observed in Figure 2a, two distinct XPS peaks, Zn 2p_3/2_ and Zn 2p_1/2_, manifest at 1045.1 eV and 1022 eV, respectively. Additionally, the presence of ZnO insulating material is confirmed by the O 1s peak at 531.5 eV. Turning our attention to Figure 2c,d, these panels reveal the Hf 4f and O 1s spectra for the HfO_x_ thin film. As can be seen in Figure 2c, the XPS peaks for Hf 4f_7/2_ and Hf 4f_5/2_ are discernible at 18.2 eV and 19.9 eV, respectively. Furthermore, the O 1s peak in Figure 2d, observed at 531.5 eV, signifies the presence of the HfO_x_ thin insulating film.

**Figure 3:**
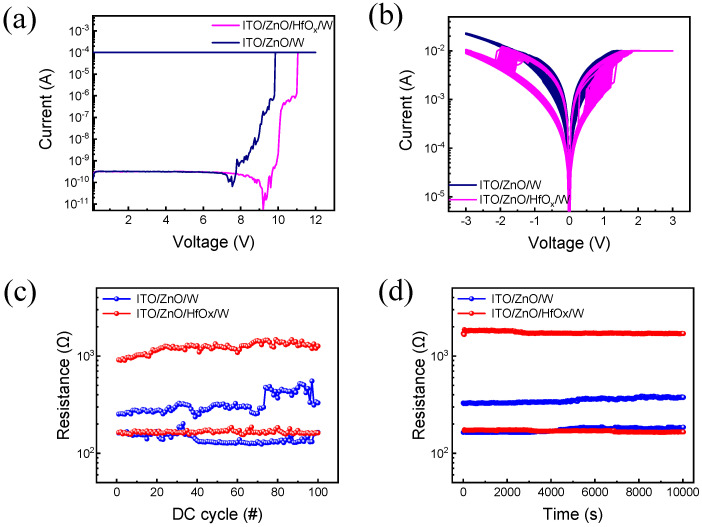
I-V characteristic of both ZnO and ZnO/HfO_x_ RRAM device. (**a**) I-V curves of forming process, (**b**) bipolar resistive switching in 100 cycles, (**c**) endurance test for 100 cycles, (**d**) retention properties for 10^4^ s.

Figure 3a illustrates the forming curves for the ZnO and ZnO/HfO_x_-based devices at a compliance current of 0.1 mA.

Before the device activation, a soft breakdown process (forming) is usually needed in the resistive switching device to accumulate defects and create an initial conductive path. As illustrated, by applying the appropriate forming voltage, the device transits from the initial resistance state (IRS) to the low resistance state (LRS). Furthermore, it is shown that the ZnO/HfO_x_ bilayered device needs more voltage for the forming process compared to the single-layered device. This may be due to the additionally deposited HfO_x_ layer, which makes the dielectric region more insulating [30]. In Figure 3b, the I-V curve is shown after 100 consecutive set and reset cycles following the forming process. To establish the devices, a voltage ramping up to 3 V was applied, while a ramped voltage of −3 V was applied to reset them. Furthermore, a compliance current of 10 mA was applied for both devices to avoid permanent breakdown [45]. Analyzing the I-V curve reveals that the ZnO/HfO_x_ RRAM exhibits improved uniformity compared to the ZnO-based RRAM. Furthermore, endurance was tested at a read voltage of 0.1 V. As demonstrated in Figure 3c, the device with HfO_x_ insertion experiences less variation. In Figure 3d, a comparison between the two devices during a retention test lasting 10^4^ s is presented. At a read voltage of 0.1 V, the ZnO/HfO_x_ device demonstrates a larger ON/OFF ratio for both HRS and LRS retention as compared to the ZnO device.

**Figure 4:**
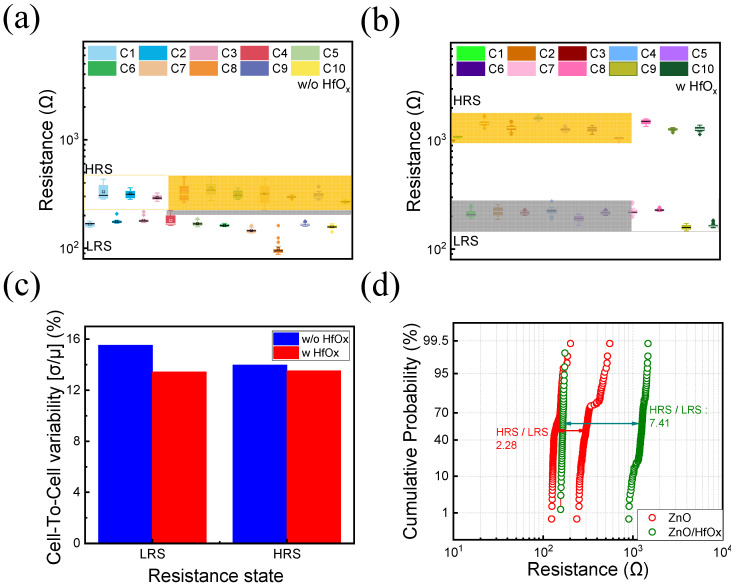
Cell-to-cell uniformity (**a**) without and (**b**) with HfO_x_ device, (**c**) cell-to-cell variability, and (**d**) cumulative probability of both ZnO and ZnO/HfO_x_ devices.

Moving forward, we conducted a comparison of the cell-to-cell uniformity of 10 randomly selected cells between the two devices, as shown in Figure 4a,b. The enhanced uniformity of the ZnO/HfO_x_ device is evident. Furthermore, Figure 4c presents a comparison of resistance distributions, revealing a reduced degree of cell-to-cell variability. In Figure 4d, a comparison of cumulative distribution functions highlights that the HRS-to-LRS ratio is 2.28 for the ZnO device and 7.41 for the ZnO/HfO_x_ device, underscoring the improved performance achieved through the introduction of HfO_x_**.** These findings emphasize the enhanced characteristics of the ZnO/HfO_x_ RRAM configuration.

**Figure 5:**
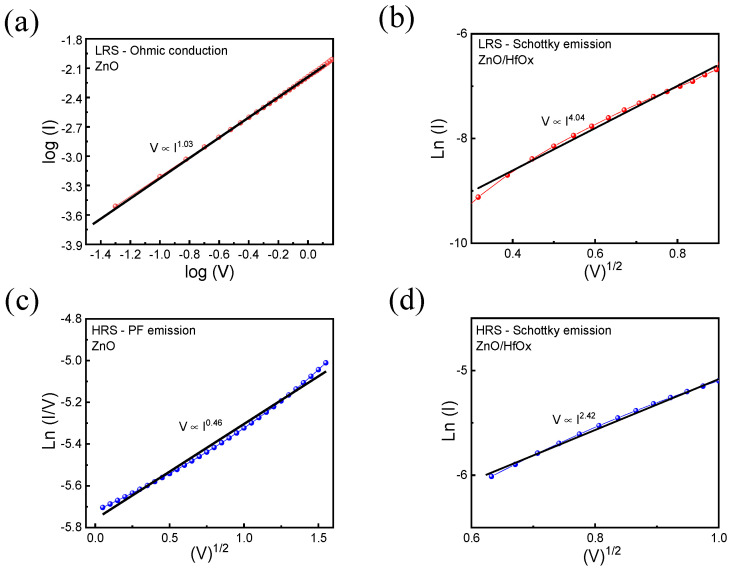
(**a**) Ohmic conduction mechanism fitting in the LRS state of ZnO device, (**b**) Schottky emission conduction mechanism fitting in the LRS state of ZnO/HfO_x_ device, (**c**) Poole–Frankel emission conduction mechanism fitting in the HRS state of ZnO device, (**d**) Schottky emission conduction mechanism fitting in the HRS state of ZnO/HfO_x_ device.

Next, we conducted an analysis of the conduction mechanisms in the two devices. For the ZnO device, the LRS conduction characteristics are represented by a dual logarithmic curve fitting, as depicted in Figure 5a, revealing a slope of 1.03. This observation indicates Ohmic conduction, which relies on resistance as a primary factor. Conversely, the HRS state demonstrates a linear relationship between ln (I/V) and sqrt (V), as shown in Figure 5c. This finding suggests the presence of the Poole–Frankel (PF) emission conduction mechanism. In the PF mechanism, thermally activated electrons surmount energy barriers located at trap sites. In contrast, for the ZnO/HfO_x_ device, Figure 5b,d illustrate that both the LRS and HRS states exhibit a linear relationship between ln(I) and sqrt(V). This pattern points towards the Schottky emission as the predominant conduction mechanism. In the Schottky emission, thermal energy aids electrons in overcoming the Schottky barrier. The equations that describe the linear relationships for Ohmic conduction, Schottky emission, and the PF mechanism are as follows:

(1) (Ohmic) I∝V

(2) $$(\mathrm{Schottky})\;\ln I\;\propto \;\frac{e\sqrt{eV/4\pi \varepsilon_{r}\varepsilon_{0}d}}{KT}$$

(3) $$(\mathrm{PF})\;\ln I/V\propto \frac{e\sqrt{eV/\pi \varepsilon_{r}\varepsilon_{0}d}}{KT}$$

where *e*, *ε_r_*, *ε*_0_, *d*, *K*, and *T* denote elementary charge, relative dielectric constant, free space permittivity, film thickness, Boltzmann’s constant, and temperature, respectively [46].

**Figure 6:**
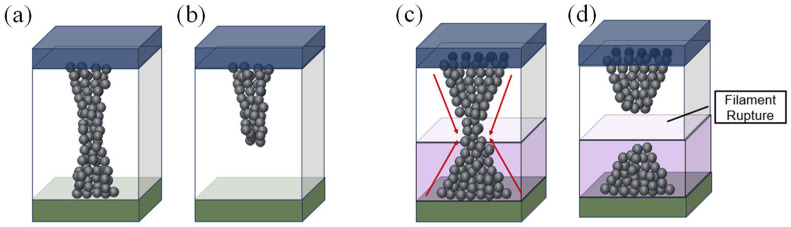
Schematic diagram of filament formation and rupture process in the RRAM device. (**a**) Set and, (**b**) reset process of ITO/ZnO/W. (**c**) Set and, (**d**) reset process of IZO/ZnO/HfO_x_/W device.

The switching mechanism of the devices is illustrated in Figure 6a–d. In the ITO/ZnO/W device, applying a positive voltage to the ITO top electrode (TE) induces the migration of oxygen ions toward the ITO side. The accumulation of oxygen vacancies within the ZnO layer facilitates the formation of a conductive filament, initiating the set process, as shown in Figure 6a. Conversely, applying a negative voltage to the TE causes oxygen ions to migrate back to the bottom electrode (BE). These ions replenish oxygen vacancies, leading to the rupture of the oxygen-deficient conductive filament near the BE. This transition characterizes the reset process, as depicted in Figure 6b.

However, due to the inherent randomness of conducting filament, the ZnO single-layer device suffers from variation of resistance states during repeated cycles [47,48]. Contrastingly, the ZnO/HfO_x_ device exhibits a distinct mechanism. Based on the previous studies reporting the conduction mechanisms in the ZnO/HfO_x_ layered structure, a conduction mechanism focusing on the formation and rupture of the conductive filament at the ZnO and HfO_x_ interface is proposed [35,36]. After the forming process, oxygen ions migrate to the TE to complete the reduction process, giving rise to an hourglass-shaped conductive filament, with its weakest point located at the interface of the ZnO and HfO_x_ layer, as shown in Figure 6c [34,35]. This filament formation is accompanied by a sudden surge in current, transitioning the device into the LRS. Conversely, applying a negative voltage to the TE triggers the reset operation. During this phase, oxygen ions migrate back to the BE, replenishing oxygen vacancies and causing the conductive filament to rupture. Thus, the weakest point, at the interface of ZnO/HfO_x_, ruptures, causing the device to return to the HRS, as depicted in Figure 6d [35]. The formation of an hourglass-shaped filament occurs when a positive voltage is applied to the ITO top electrode. Oxygen vacancies from the ZnO layer migrate through the HfO_x_ layer to the bottom W electrode. In effect, the oxygen vacancies in the HfO_x_ layer transform it into a virtual electrode. These vacancies could play a catalytic role, essential for initiating the emergence of the conductive filament within ZnO. The resistive switching (RS) mechanism is regulated by the constriction imposed by the matrix layer, governing the filament’s rupture and recovery at the ZnO/HfO_x_ interface and ensuring precise switching uniformity [34]. Notably, the primary site of conductive filament formation and rupture occurs at the contact interface between ZnO and HfO_x_ layers [49,50].

**Figure 7:**
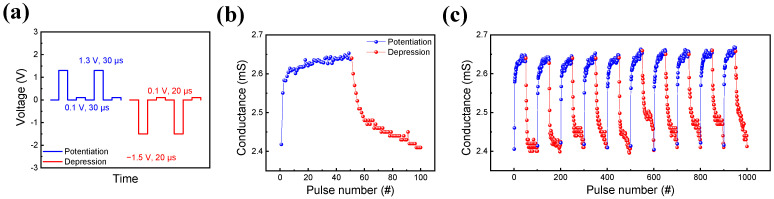
(**a**) Pulse schematic about potentiation and depression, (**b**) potentiation and depression characteristics, (**c**) potentiation and depression characteristics under 10 cycles of an identical pulse train.

The feasibility of pulse response testing for the device is demonstrated by its application in neuromorphic devices. The pulse trains for both potentiation and depression are depicted in Figure 7a.

To acquire potentiation and depression, a sequence of 50 set pulses and reset pulses with an amplitude of 1.3 V and −1.5 V was applied with a pulse of 30 μs and 20 μs. Additionally, to observe the conductance change through the applied pulse, a pulse of 0.1 V followed the consecutive pulse trains. The interval time between all pulse trains was consistently maintained at 10 μs. The outcomes of the pulse response are presented in Figure 7b, revealing a gradual increase and decrease in conductance. Further insight is provided in Figure 7c, which displays 10 cycle graphs for both potentiation and depression. These graphs underscore the consistent conductance levels observed across each cycle, affirming the device’s reliability as a neuromorphic element [51]. This specific component’s conductance can be employed as a weight in neural networks.

**Figure 8:**
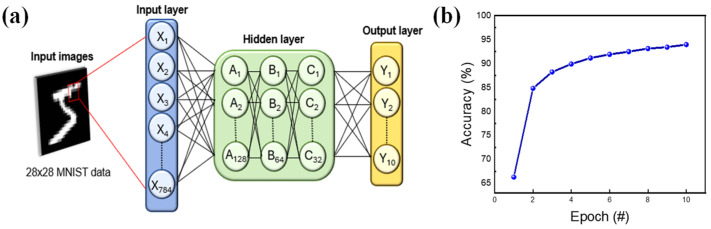
(**a**) Schematic diagram of MNIST neural networks for pattern recognition. (**b**) Implementation of recognition accuracy using MNIST.

To validate its performance, deep neural network (DNN)-based handwritten digit recognition accuracy was assessed using the MNIST database with Python in Google Colab. As shown in Figure 8a, the MNIST dataset encompasses 28 × 28-pixel grayscale images of handwritten digits, resulting in a neural network architecture with 784 input neurons and 10 output neurons. Additionally, the incorporated hidden layer consists of three hidden layers with 128, 64, and 32 nodes to enhance accuracy. Each digit of 28 × 28-pixel grayscale images has values ranging from 0 to 1. The corresponding white and black pixels of these two-digit data were gathered, forming a number image. For the input dataset, the potentiation and depression curves from Figure 7b were used and converted into an input image. The conversion followed the increase and the decrease of conductance value, making the value of the pixel change linearly compared to the previous image. Then, the number of images was inserted into the DNN-based pattern recognition system, where nodes of each layer calculated the accuracy of the handwritten image, as illustrated in Figure 8b. Impressively, an accuracy rate of 93.96% was achieved, substantiating the device’s utility as a robust element within neuromorphic systems [52].

**Figure 9:**
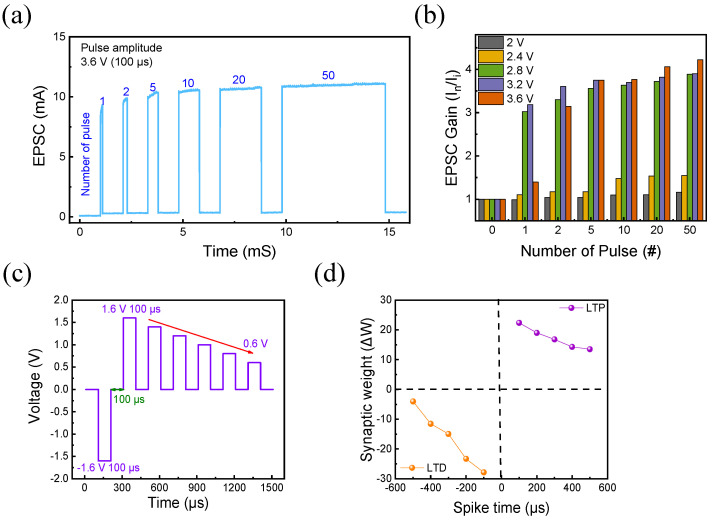
(**a**) EPSC response at 100 μs pulse width while increasing the number of pulses from 1 to 50 at a constant pulse amplitude of 3.6 V, (**b**) EPSC gain when the pulse amplitude is increased from 2 V to 3.6 V while the number of pulses increases, (**c**) STDP schematic, (**d**) result of STDP.

The excitatory post-synaptic current (EPSC) responses in the ITO/ZnO/HfO_x_/W memristor are depicted in Figure 9a,b. In Figure 9a, the post-synaptic response of the ITO/ZnO/HfO_x_/W device is shown when a consistent pulse amplitude of 3.6 V is applied with a pulse width of 100 μs, while the number of pulses is varied from 1 to 50. It is evident that as the number of pulses (input spikes) increases, the EPSC current value (output spike) also increases, indicating a positive correlation. Furthermore, Figure 9b displays the EPSC gain when the pulse amplitude is varied from 2 V to 3.6 V in increments of 0.4 V, all with a constant 100 μs pulse width. The EPSC gain is calculated using Equation (4) [53], where I_n_ and I_i_ represent the current after the last pulse and the initial current of the device, respectively. Notably, at a constant pulse amplitude, an increasing number of pulses leads to an augmented EPSC gain. This observation suggests that the increase in EPSC with a greater number of pulses mimics the synaptic response seen in biological synapses due to the relaxation process [54,55]. 

Finally, for further emulation, the Hebbian learning rule, also known as spike-timing-dependent plasticity (STDP), was implemented. STDP is a brain function of connection between the pre- and post-synapses of the brain. Between each synapse, information migrates through electrical stimuli at high speed. The strength of the synapse connection depends on the differing spike firing times of the pre- and post-synapses, resulting in different memory activities. This temporal disparity between pre-synaptic and post-synaptic neuron firings becomes evident through the gradual resistive switching of the device [56,57]. In this context, the conductive filament connecting the TE and BE simulates the roles of pre-synaptic and post-synaptic neurons in a human neural network [58,59]. To observe this phenomenon, pulse pairs are administered to each electrode. Figure 9c illustrates the pulse train applied to the terminal electrode, integrating a timing gap denoted as Δt between the two electrodes. The pulse interval remains fixed at 100 μs. During potentiation, the pre-spike precedes the post-spike. Conversely, for depression, a negative pulse train causes the post-spike to precede the pre-spike. Equation (5) expresses the synaptic weight, where *G_i_* represents the initial conductance before the pulse pair, and *G_f_* represents the conductance following the pulse pair. As shown in Figure 9d, sequential alterations in synaptic weights occur due to the time differences between spikes. This experimental setup effectively demonstrates how the gradual resistive switching of the device mirrors the fundamental principles of STDP, showcasing the potential applicability of the device in emulating synaptic plasticity within neuromorphic systems.

(4) $$EPSCgain=I_{n}/I_{i}$$

(5) $$∆W=\frac{G_{f}-G_{i}}{G_{i}}\times 100\;(\%)$$

## 4. Conclusions

This study has compellingly established the supremacy of ZnO/HfO_x_-based RRAM over its ZnO-based counterpart. The meticulous control of filament formation and rupture at the interface has led to remarkable advancements in various domains, including heightened uniformity in DC I-V bipolar switching, a substantial amplification of the ON/OFF ratio, steadfast endurance (>10^2^), and retention (>10^4^ s). Notably, this precise control facilitates pivotal synaptic functionalities like potentiation and depression. Various synapse functions were mimicked by precisely controlling the pulse scheme application to the device. By applying a controlled number of pulses with varied pulse amplitudes, the EPSC data were gained. Next, the synapse connection was tested through a Hebbian learning rule, STDP. Additionally, by applying a sequential pulse scheme of set and reset pulses, the gradual change of conductance, potentiation, and depression was achieved and repeated for 10 cycles to ensure its uniformity and repeatability. Furthermore, by using the previous potentiation and depression curve, the DNN-based MNIST PRS was implemented and tested to seek its number recognition ability. Collectively, we believe that the ITO/ZnO/HfO_x_/W device we fabricated in this paper showed affirmable potential in its neuromorphic application due to its various synapse-emulating functions. As the field of neuromorphic computing advances, this device shows the potential to make a significant contribution to the evolution of efficient and sophisticated neural network systems.

## Data Availability

Not applicable.

## References

[1] Wu J., Shen Y.L., Reinhardt K., Szu H., Dong B. (2013). A Nanotechnology Enhancement to Moore’s Law. Appl. Comput. Intell. Soft Comput..

[2] Fong S.W., Neumann C.M., Wong H.S.P. (2017). Phase-Change Memory—Towards a Storage-Class Memory. IEEE Trans. Electron. Devices.

[3] Sarwat S.G. (2017). Materials science and engineering of phase change random access memory. Mater. Sci. Technol..

[4] Patriarca R., Gravio G.D., Woltjer R., Costantino F., Praetourius G., Ferreira P., Hollnagel E. (2020). Framing the FRAM: A literature review on the functional resonance analysis method. Saf. Sci..

[5] Eshita T., Wang W., Nomura K., Nakamura K., Saito H., Yamaguchi H., Mihara S., Hikosaka Y., Kataoka Y., Kojima M. (2018). Development of Highly Reliable Ferroelectric Random Access Memory and Its Internet of Things Applications. Jpn. J. Appl. Phys..

[6] Zhao W.S., Devolder T., Lakys Y., Klein J.O., Chappert C., Mazoyer P. (2011). Design Considerations and Strategies for High-Reliable STT-MRAM. Microelectron. Reliab..

[7] Khvalkovskiy A.V., Apalkov D., Watts S., Chepulskii R., Beach R.S., Ong A., Tang X., Driskill-Smith A., Butler W.H., Visscher P.B. (2013). Basic Principles of STT-MRAM Cell Operation in Memory Arrays. J. Phys. D Appl. Phys..

[8] Gurme S.T., Dongale T.D., Surwase S.N., Kumbhar S.D., More G.M., Patil V.L., Patil P.S., Kamat R.K., Jadhav J.P. (2018). An Organic Bipolar Resistive Switching Memory Device Based on Natural Melanin Synthesized from *Aeromonas* sp. SNS Phys. Status Solidi A Appl. Mat..

[9] Pawar P.S., Tikke R.S., Patil V.B., Mullani N.B., Waifalkar P.P., Khot K.V., Teli A.M., Sheikh A.D., Dongale T.D. (2017). A Low-Cost Copper Oxide Thin Film Memristive Device Based on Successive Ionic Layer Adsorption and Reaction Method. Mater. Sci. Semicond. Process..

[10] Meena J.S., Sze S.M., Chand U., Tseng T.Y. (2014). Overview of Emerging Nonvolatile Memory Technologies. Nanoscale Res. Lett..

[11] Hwang C.S. (2015). Prospective of Semiconductor Memory Devices: From Memory System to Materials. Adv. Electron. Mater..

[12] Zahoor F., Azni Zulkifli T.Z., Khanday F.A. (2020). Resistive Random Access Memory (RRAM): An Overview of Materials, Switching Mechanism, Performance, Multilevel Cell (MLC) Storage, Modeling, and Applications. Nanoscale Res. Lett..

[13] Banerjee W. (2020). Challenges and Applications of Emerging Nonvolatile Memory Devices. Electronics.

[14] Ventra M.D., Pershin Y.V. (2011). Memory Materials: A Unifying Description. Mater. Today.

[15] Pan F., Gao S., Chen C., Song C., Zeng F. (2014). Recent Progress in Resistive Random Access Memories: Materials, Switching Mechanisms, and Performance. Mater. Sci. Eng. R Rep..

[16] Khot A.C., Dongale T.D., Nirmal K.A., Sung J.H., Lee H.J., Nikam R.D., Kim T.G. (2022). Amorphous Boron Nitride Memristive Device for High-Density Memory and Neuromorphic Computing Applications. ACS Appl. Mater. Interfaces.

[17] Nirmal K.A., Nhivekar G.S., Khot A.C., Dongale T.D., Kim T.G. (2022). Unraveling the Effect of the Water Content in the Electrolyte on the Resistive Switching Properties of Self-Assembled One-Dimensional Anodized TiO2 Nanotubes. J. Phys. Chem. Lett..

[18] Jeon D.S., Dongale T.D., Kim T.G. (2021). Low Power Ti-Doped NbO2-Based Selector Device with High Selectivity and Low OFF Current. J. Alloys Compd..

[19] Patil A.R., Dongale T.D., Kamat R.K., Rajpure K.Y. (2021). Spray Pyrolysis Deposited Iron Tungstate Memristive Device for Artificial Synapse Application. Mater. Today Commun..

[20] Park J., Kim S. (2022). Improving Endurance and Reliability by Optimizing the Alternating Voltage in Pt/ZnO/TiN RRAM. Results Phys..

[21] Kwon O., Shin J., Chung D., Kim S. (2022). Energy Efficient Short-Term Memory Characteristics in Ag/SnOx/TiN RRAM for Neuromorphic System. Ceram. Int..

[22] Ju D., Kim J.H., Kim S. (2023). Highly Uniform Resistive Switching Characteristics of Ti/TaOx/ITO Memristor Devices for Neuromorphic System. J. Alloys Compd..

[23] Park J., Ryu H., Kim S. (2021). Nonideal Resistive and Synaptic Characteristics in Ag/ZnO/TiN Device for Neuromorphic System. Sci. Rep..

[24] Slesazeck S., Mikolajick T. (2019). Nanoscale resistive switching memory devices: A review. Nanotechnology.

[25] Kang D., Jang J.T., Park S., Ansari M.H.R., Bae J.H., Choi S.J., Kim D.M., Kim C., Cho S., Kim D.H. (2021). Threshold-Variation-Tolerant Coupling-Gate α-IGZO Synaptic Transistor for More Reliably Controllable Hardware Neuromorphic System. IEEE Access.

[26] Zhu X., Su W., Liu Y., Hu B., Pan L., Lu W., Zhang J., Li R.W. (2012). Observation of Conductance Quantization in Oxide-Based Resistive Switching Memory. Adv. Mater..

[27] Wang Y., Liu Q., Long S., Wang W., Wang Q., Zhang M., Zhang S., Li Y., Zuo Q., Yang J. (2010). Investigation of Resistive Switching in Cu-Doped HfO_2_ Thin Film for Multilevel Non-Volatile Memory Applications. Nanotechnology.

[28] Yang J., Ryu H., Kim S. (2021). Resistive and Synaptic Properties Modulation by Electroforming Polarity in CMOS-Compatible Cu/HfO_2_/Si Device. Chaos Solitons Fractals.

[29] Huang Y., Shen Z., Wu Y., Wang X., Zhang S., Shi X., Zeng H. (2016). Amorphous ZnO Based Resistive Random Access Memory. RSC Adv..

[30] Kumar D., Chand U., Siang L.W., Tseng T.Y. (2020). High-Performance TiN/Al_2_O_3_/ZnO/Al_2_O_3_/TiN Flexible RRAM Device with High Bending Condition. IEEE Trans. Electron. Devices.

[31] Kim D.C., Lee M.J., Ahn S.E., Seo S., Park J.C., Yoo I.K., Baek I.G., Kim H.J., Yim E.K., Lee J.E. (2006). Improvement of Resistive Memory Switching in NiO Using IrO_2_. Appl. Phys. Lett..

[32] Kao M.C., Chen H.Z., Chen K.H., Shi J.B., Weng J.H., Chen K.P. (2020). Resistive switching behavior and optical properties of transpaent Pr-doped ZnO based resistive random access memory. Thin Solid Films.

[33] Simanjuntak F.M., Panda D., Wei K.H., Tseng T.Y. (2016). Status and Prospects of ZnO-Based Resistive Switching Memory Devices. Nanoscale Res. Lett..

[34] Jain N., Sharma S.K., Kumawat R., Jain P.K., Kumar D., Vyas R. (2022). Resistive Switching, Endurance and Retention Properties of ZnO/HfO_2_ Bilayer Heterostructure Memory Device. Micro Nanostruct..

[35] Hsieh W., Lam K., Chang S. (2015). Bipolar Ni/ZnO/HfO_2_/Ni RRAM with multilevel characteristic by different reset bias. Mater. Sci. Semicond. Process.

[36] Zhang W., Guo Z., Dai Y., Lei J., Wang J., Hu F. (2023). Effects of stacking sequence and top electrode configuration on switching behaviors in ZnO-HfO_2_ hybrid resistive memories. Ceram. Int..

[37] Chen S., Chen H., Lai Y. (2022). Reproducible Non-Volatile Multi-State Storage and Emulation of Synaptic Plasticity Based on a Copper-Nanoparticle-Embedded HfOx/ZnO Bilayer with Ultralow-Switching Current and Ideal Data Retention. Nanomaterials.

[38] Wu S.J., Wang F., Zhang Z.C., Li Y., Han Y.M., Yang Z.C., Zhao J.S., Zhang K.L. (2018). High Uniformity and Forming-Free ZnO-Based Transparent RRAM with HfOx Inserting Layer. Chin. Phys. B.

[39] Lai Y., Zeng Z., Liao C., Cheng S., Yu J., Zheng Q., Lin P. (2016). Ultralow Switching Current in HfOx/ZnO Bilayer with Tunable Switching Power Enabled by Plasma Treatment. Appl. Phys. Lett..

[40] Zhang X., Liu S., Zhao X., Wu F., Wu Q., Wang W., Cao R., Fang Y., Lv H., Long S. (2017). Emulating Short-Term and Long-Term Plasticity of Bio-Synapse Based on Cu/a-Si/Pt Memristor. IEEE Electron Device Lett..

[41] Querlioz D., Bichler O., Vincent A.F., Gamrat C. (2015). Bioinspired Programming of Memory Devices for Implementing an Inference Engine. Proc. IEEE.

[42] Huang P., Li Z., Dong Z., Han R., Zhou Z., Zhu D., Liu L., Liu X., Kang J. (2019). Binary Resistive-Switching-Device-Based Electronic Synapse with Spike-Rate-Dependent Plasticity for Online Learning. ACS Appl. Electron. Mater..

[43] Lecun Y., Bottou E., Bengio Y., Haffner P. (1998). Gradient-Based Learning Applied to Document Recognition. Proc. IEEE.

[44] Lee Y., Park J., Chung D., Lee K., Kim S. (2022). Multi-Level Cells and Quantized Conductance Characteristics of Al_2_O_3_-Based RRAM Device for Neuromorphic System. Nanoscale Res. Lett..

[45] Chang Y.F., Chen P.Y., Fowler B., Chen Y.T., Xue F., Wang Y., Zhou F., Lee J.C. (2012). Understanding the Resistive Switching Characteristics and Mechanism in Active SiOx-Based Resistive Switching Memory. J. Appl. Phys..

[46] Yuan F.Y., Deng N., Shih C.C., Tseng Y.T., Chang T.C., Chang K.C., Wang M.H., Chen W.C., Zheng H.X., Wu H. (2017). Conduction Mechanism and Improved Endurance in HfO_2_-Based RRAM with Nitridation Treatment. Nanoscale Res. Lett..

[47] Li T., Yu H., Chen S.H.Y., Zhou Y., Han S.T. (2020). The Strategies of Filament Control for Improving the Resistive Switching Performance. J. Mater. Chem. C.

[48] Zhu X., Shang J., Li R.W. (2012). Resistive switching effects in oxide sandwiched structures. Front. Mater. Sci..

[49] Kumar D., Kalaga P.S., Ang D.S. (2020). Visible Light Detection and Memory Capabilities in MgO/HfO Bilayer-Based Transparent Structure for Photograph Sensing. IEEE Trans. Electron Devices.

[50] Zhang R., Huang H., Xia Q., Ye C., Wei X., Wang J., Zhang L., Zhu L.Q. (2019). Role of Oxygen Vacancies at the TiO_2_/HfO_2_ Interface in Flexible Oxide-Based Resistive Switching Memory. Adv. Electron. Mater..

[51] Wang Q., Niu G., Roy S., Wang Y., Zhang Y., Wu H., Zhai S., Bai W., Shi P., Song S. (2019). Interface-Engineered Reliable HfO_2_-Based RRAM for Synaptic Simulation. J. Mater. Chem. C.

[52] Zhang S.R., Zhou L., Mao J.Y., Ren Y., Yang J.Q., Yang G.H., Zhu X., Han S.T., Roy V.A.L., Zhou Y. (2019). Artificial Synapse Emulated by Charge Trapping-Based Resistive Switching Device. Adv. Mater. Technol..

[53] Qin K., Zhou F., Wang J., Chen C., Wang X., Guo S., Zhao Y., Pei L., Zhen P.D., Ye S.P. (2020). Anisotropic signal processing with trigonal selenium nanosheet synaptic transistors. ACS Nano..

[54] Lan S., Zhong J., Chen J., He W., He L., Yu R., Chen G., Chen H. (2021). An optoelectronic synaptic transistor with efficient dual odulation by light illumination. J. Mater. Chem. C.

[55] Wei H., Han H., Guo K., Yu H., Gong J., Ma M., Ni Y., Feng J., Xu Z., Xu W. (2021). Artificial synapses that exploit ionic modulation for perception and integration. Mater. Today Phys..

[56] Ryu H., Choi J., Kim S. (2020). Voltage Amplitude-Controlled Synaptic Plasticity from Complementary Resistive Switching in Alloying HfOx with AlOx-Based RRAM. Metals.

[57] Kim C., Lee Y., Kim S., Kang M., Kim S. (2023). Diverse synaptic weight adjustment of bio-inspired ZrOx-based memristors for neuromorphic system. Mater. Sci. Semicond. Process..

[58] Wang W., Pedretti G., Milo V., Carboni R., Calderoni A., Ramaswamy N., Spinelli A.S., Ielmini D. (2018). Learning of spatiotemporal patterns in a spiking neural network with resistive switching synapses. Sci. Adv..

[59] Yu S., Yi W., Jeyasingh R., Kuzum D., Philip W.H.S. (2011). An Electronic Synapse Device Based on Metal Oxide Resistive Switching Memory for Neuromorphic Computation. IEEE Trans. Electron Devices.

